# Contribution of Two Different Packaging Material to Microbial Contamination of Peaches: Implications in Their Microbiological Quality

**DOI:** 10.3389/fmicb.2016.00938

**Published:** 2016-06-16

**Authors:** Francesca Patrignani, Lorenzo Siroli, Fausto Gardini, Rosalba Lanciotti

**Affiliations:** Department of Agricultural and Food Sciences, University of BolognaCesena, Italy

**Keywords:** packaging, cardboard, reusable plastic container, vegetables, quality, shelf-life

## Abstract

**Aim:** Aim of this work was understanding the microbial transfer dynamics from packaging to packed peaches in relation to the packaging used.

**Method and Results:** A challenge test was performed, inoculating *Escherichia coli, Pseudomonas* spp. and *Saccharomyces cerevisiae* on cardboards and RPC (Reusable Plastic Containers), and monitoring their cell loads on fruits according to a probabilistic model and a Response Surface Methodology (RSM) in relation to several independent variables (number of fruit lesions, fruit temperature storage and commercialization time). The data recorded on packed peaches for *Pseudomonas* and *S. cerevisiae* were modeled to fit the second order model to study the main, interactive and quadratic effects of the independent variables on the cell loads of target microorganisms as well as on the shelf-life of the fruits in relation to packaging material used. The data collected for *E. coli* were codified as presence (1) or absence (0) and modeled with a logistic regression analysis to assess the probability of *E. coli* transferring from packaging to fruits in relation to the adopted variables. The data showed a higher contamination frequency of the fruits packed in plastic than in cardboard. Increasing the storage temperature and the number of lesions, the probability of transferring of *E. coli* from packaging materials to fruits increased, independently on commercialization time or packaging used. For *Pseudomonas*, the contamination levels detected on fruits packaged in plastic were significantly higher compared to those found on fruits packed in cardboard, independently on the considered variables. The polynomial equations showed the *S. cerevisiae* cell loads of fruits stored in plastic was positively affected by the quadratic term of temperature.

**Conclusions:** the use of cardboard, compared to plastic, can significantly reduce the potential of microbial transferring from packaging to fruits. The probabilistic and kinetic models used showed a higher microbiological qualities of peaches stored in cardboard boxes, independently on the independent variables considered. The best performances of cardboard, compared to plastic, was probably due to its capability to entrap microbial cells.

**Significance and Impact:** cardboard reduces fruit contamination and increases their shelf-life with positive fallouts on fruit shelf-life and all the logistic and distribution chain.

## Introduction

The adhesion and persistence of microorganisms to the surfaces can spread pathogens and spoilage microorganisms to foods, influencing their shelf-life and safety (Barnes et al., 1999; Bae et al., 2012). Several studies have showed the ability of microorganisms to attach to all the surfaces commonly found in the food processing environment, such as stainless steel, polystyrene, rubber, glass, wood and so on (Czechowski, 1990; Mafu et al., 1990; Krysinski et al., 1992; Suárez et al., 1992; Barnes et al., 1999; Siroli et al., 2014). Additionally, if microorganisms remain on a given surface for a relatively long time, they can multiply and, eventually, form biofilms (Uhlich et al., 2006). Although no literature reports are available on the survival of microorganisms on packaging materials, several studies showed that various foodborne pathogens, including *Escherichia coli* and *Listeria monocytogenes*, can survive on utensils and equipment surfaces for hours or days (Kusumaningrum et al., 2003; Wilks et al., 2005, 2006; Martinon et al., 2012).

Microbial cross-contamination refers to the transfer, direct or indirect, of microorganisms (bacteria, virus, parasites, or fungi) from a contaminated item to a non-contaminated one (Minnesota Department of Health, 2007). In food, cross contamination of foodborne pathogens is a major concern since it increases the health risk for humans due to the intake of contaminated food. Otherwise, cross-contamination of foodborne pathogens from inert surfaces to foods is well documented (Kusumaningrum et al., 2003; Lin et al., 2006; Wilks et al., 2006; De Candia et al., 2015; Erickson et al., 2015).

On the other hand, fresh produce have been associated in several outbreaks caused by *E. coli* O157:H7, *Salmonella* spp. and *L. monocytogenes* (Alegre et al., 2010; Scallan et al., 2011; Oliveira et al., 2012; Siroli et al., 2014). According to EFSA (2013), these products are involved in more than 5% of food borne illness in Europe. Also the USA Centre for Disease Control and Prevention (CDC) clearly showed the fresh produce as a source of contamination leading to food borne illnesses. In fact, pathogens, eventually introduced during the production chain, may remain until the product consumption due to the lacking of treatments able to eradicate the microbial cells. The interruption of cold chain during distribution, sale and home storage determine rapid deterioration of these products due to the growth of spoilage microorganisms present on fruit and vegetable. To increase the limited shelf-life of fresh produce the tendency is to pack unripe fruit and vegetable characterized by lower sensory features compared to ripe fruits. Consequently, controlling the permanence of microorganisms on surfaces, including packaging materials, is fundamental in reaching food safety standards and improving the overall quality (i.e., texture, flavor, aroma) and shelf-life of fresh produce. The literature data on the contamination levels of packaging materials are few and fragmented. However, they demonstrated that packaging materials can be contaminated by spoilage and pathogenic microorganisms (Suominen et al., 1997). The cell loads normally detected for mesophylic aerobic bacteria ranged between 10^3^ and 10^6^ cfu/cm^2^ for packages of recycled materials and between 10^2^ and 10^5^ cfu/cm^2^ for products based on virgin fibers (Suominen et al., 1997). The wide variability is mainly due to the differences in physico-chemical features of packaging materials but also in logistic such as transportation. The few literature data show that spore-forming bacteria (belonging to the genera *Bacillus, Geobacillus, Alicyclobacillus*, and *Clostridium*) and molds (belonging mainly to the species *Aspergillus niger, A. cinnamomeus*, and *Cladosporium herbarum*) prevail on packaging microbiota. They are widespread microorganisms, resistant to adverse environmental conditions and endowed with high spoilage potential (Binderup et al., 2002; Turtoi and Nicolau, 2007). However, also yeast and other spoilage bacteria can be present on packaging materials. To avoid and/or minimize this issue, the use of appropriate packaging is essential, since it acts as a barrier that can protect fresh food from contamination (Campos et al., 2014). The importance of paper-based materials has been already recognized for many years. The greatest benefit of these ones in comparison to plastic materials is their comparatively minimal impact on our environment and biodegradability (Levi et al., 2011; Hladíková et al., 2015). However, although the Regulation (EC) No 852/2004 on materials and articles intended to come into contact with food stipulates that “the packaging must not be a source of food contamination,” understanding the real contribution of the packaging material in product contamination is not very simple due to the impossibility to establish “*a priori*” the level of the naturally occurring fruit and packaging microflora. In addition, the microbial survival, growth or death on the packaging materials, and consequently their role in cross contamination of packed fruits, are affected by environmental conditions, including storage temperature, relative humidity and nutrient availability (Siroli et al., 2014; De Candia et al., 2015; Erickson et al., 2015). Also the growth potential of the microorganisms on fruit surface is affected by the intrinsic features of fruit species and variety (i.e., specific surface features, acidity, sugar content and so on), by the ripening and by the presence of wounds and exudates (Heaton and Jones, 2008). In this framework, aim of the current research was to evaluate the role of the packaging material in the cross-contamination of packed peaches in relation to some environmental conditions and fruit quality. To reach this goal and to understand the dynamics of microbial transfer from packaging to packed fresh peaches, a challenge test was performed. In particular, *E. coli, Pseudomonas* spp., and *Saccharomyces cerevisiae* were inoculated on two different types of packaging, such as cardboards and reusable plastic containers (RPC) and their cell loads on the packed fruits during the storage were monitored. *Pseudomonas* spp. and *S. cerevisiae* were used in the present study as target microorganisms because frequently involved in fresh produce spoilage and recorded at high cell loads in spoiled fruits mainly in correspondence of rotten spots (Hyun et al., 2015). To study the effects of storage temperature and time of storage during the commercialization, as well as the fruit quality, chosen as independent variables, on the transferring of target microorganisms from packaging materials to fruits, a multi-variable experimental design was set-up. To evaluate the relationships among the considered independent variables and the probability of transferring of *E. coli* from packaging materials to stored fruits, a logit model was used. In fact, logistic regression is a useful tool in predictive food microbiology to determine the food safety in relation to food composition, process or storage variables (Zhao et al., 2001; Belletti et al., 2007). In addition, the Response Surface Methodology (RSM) was used to study the main, interactive and quadratic effects of the independent variables on the cell loads of *Pseudomonas* spp. and *S. cerevisiae* as well as on the shelf-life of the fruits in relation to packaging material used.

## Materials and methods

### Microorganisms

*Escherichia coli* E555, *Pseudomonas* spp. and *Saccharomyces cerevisiae* Spa, belonging to the Department of Agricultural and Food Sciences (DISTAL, University of Bologna), were employed in this study. The spoilage strains were isolated from spoiled peaches.

The stock cultures of *E. coli* and *Pseudomonas* spp. were maintained in BHI broth (Oxoid, Basingstoke, UK) while *S. cerevisiae* was stocked in YPD broth (Oxoid, Basingstoke, UK). All contained sterile glycerol (20%v/v) and were stored at −70°C. Fresh cultures of each strain were obtained by two consecutive passages of a 1%(v/v) inoculums of the frozen stocks in appropriate broths and incubation conditions.

### Packaging and fruits

The packaging used in this research were cardboard (60 cm × 40 cm) and RPC (60 cm × 40 cm). The cardboard was certified by Bestack and purchased by Ghelfi Ondulati S.p. A (Cesena, Italy) while RPC boxes were bought at a local fruit and vegetable gross market (Cesena, Italy) and sanitized before using. The peaches (var. MAYCREST) were purchased by a local distributor (Cesena, Italy). The packaging and peaches were checked for the initial contamination levels for coliforms, yeasts and *Pseudomonas* spp.

### Set-up of the experimental plan

In order to understand the role of the packaging material in the cross-contamination of packed peaches in relation to some environmental conditions and fruit quality, a Central Composite Design (3 independent variables and 5 levels) (CCD) was performed considering temperature of fruit storage, commercialization time (storage time during commercialization) and number of fruit lesions as independent variables (Table 1) according to Box et al. (1978). In addition to the 17 runs, CCD was reinforced by the addition of 3 additional combinations (18, 19, 20) to allow a better prediction at the lowest and highest values of the three independent variables considered according to Belletti et al. (2010). Peaches were washed with tap water, surface disinfected by dipping for 1 min in 1% (w/v) of sodium hypochlorite (NaOCl) solution, rinsed with sterilized water and then air-dried. Also RPCs were decontaminated before inoculation following the same procedures used for peaches. In contrast, the corrugated boxes were produced by Ghelfi just before the set-up of the trials and stocked before usage avoiding re-contamination through the protection of a proper film. In order to understand the dynamic of microbial transferring from packaging material to packed peaches, RPC and corrugated were deliberately inoculated with the target microorganisms at level of 2 log cfu/cm^2^ for *E. coli* and between 3 and 4 log cfu/cm^2^ for *Pseudomonas* spp. and *S. cerevisiae*. The target microorganisms vehicle in ringer solution (20 ml for each box) were sprayed on the box surfaces and let dry at room temperature. The same inoculation level was used for corrugated and RPC boxes. Following, all the boxes were filled with the peaches that presented a different number of lesions and stored at the temperature established by the experimental plan (Table 1). At the times established by the experimental plan, the transfer of the target microorganisms was evaluated. For each run of the experimental plan, 20 fruits were analyzed. For *Pseudomonas* spp. and *S. cerevisiae*, for each run considered, the fruits were analyzed also during a further 48–72 h of storage, in addition to the times fixed by the experimental plan, in order to collect from 4 to 6 additional points to use for primary growth model fitting and estimating the time necessary to reach 7 log cfu/fruit. The data collected over the whole storage were used to evaluate the time necessary to reach an arbitrary threshold of 7 log cfu/fruit (see Section Data Analysis). The storage was prolonged further 48 h for the samples stored at 19 and 24°C, and of 72 h for the samples stored at 4, 9, and 14°C. The different storage times, in relation to temperature, were due to the effect of temperature on fruit quality. So the storage period considered to evaluate the time necessary to reach an arbitrary threshold of 7 log cfu/fruit ranged between 86.5 and 144.5 h.

**Table 1:** Independent variable levels adopted in the Central Composite Design^*^.

**Run**	**Temperature (°C)**	**Commercialization time (h)**	**Number of lesion (n)**
1	9	29	1
2	9	58	1
3	9	29	3
4	9	58	3
5	19	29	1
6	19	58	1
7	19	29	3
8	19	58	3
9	14	43.5	2
10	14	43.5	2
11	14	14.5	2
12	14	72.5	2
13	14	43.5	0
14	14	43.5	4
15	4	43.5	2
16	24	43.5	2
17	14	43.5	2
18	19	58	0
19	9	58	0
20	9	29	0

* *CCD was reinforced by the addition of 3 additional combinations (18, 19, 20) to allow a better prediction at the lowest and highest values of the three independent variables considered*.

### Microbiological analysis

At the time established, 20 fruits for each run were randomly taken and analyzed. Each fruit was washed with 30 ml of ringer solution (0.9% NaCl). The peaches were maintained in agitation for 5 min and following the washing water was analyzed. To check the microbiological quality of the packaging, after decontamination and/or before inoculation of the target microorganisms, a superficial swab was performed analyzing 10 cm^2^.

*E. coli* was found by using VRBA (Violet Red Bile Agar, Oxoid), added with MUG (4-methylumbelliferyl-β-D-glucuronide); *Pseudomonas* was detected on *Pseudomonas* agar base (Oxoid) while *S. cerevisiae* on YPD agar according to the procedures described in Lanciotti et al. (2004a) and Siroli et al. (2014). The plates were then incubated at the optimal temperature for each considered microorganism. In particular, for *E. coli*, the plates were incubated at 37°C for 24 h while for *Pseudomonas* and yeasts they were incubated at 27°C for 48 h.

### Data analysis

For *E. coli*, the data obtained was codified as presence (1) or absence (0). On the basis of the obtained results (1 or 0), a logistic regression analysis (Hosmer and Lemeshow, 1989) was carried out using the statistical package SPSS v. 19 (SPSS Inc., Chicago, Ill., U.S.A.) in order to assess the probability (P) of *E. coli* transfer from packaging to fruits in relation to the adopted variables. The generic model used for multiple logistic regression was:

$$\begin{array}{l} P=\frac{e^{\left(a+bx\right)}}{1+e^{\left(a+bx\right)}} \end{array}$$

According to Hosmer and Lemeshow (2000), the different relationships between the logit and the continuous independent variables can assume a linear, quadratic, and other non-linear form.

In this case, g(x) corresponds to the equation:

$$\begin{array}{l} g\;\left(\text{x}\right)=\beta 0+\sum \text{βi xi }+\;\sum \text{βi i x}2\;+\sum \text{βi j xi xj} \end{array}$$

where the covariates (i…j) were, in this case, the independent variables. According to Hosmer and Lemeshow (2000), the logistic equation can be linearized, and, in this case, it can be transformed into:

$$\begin{array}{l} Logit\left(P\right)\;=\text{ ln }\left(\frac{P}{1-P}\right)\;=\;a\;+\;bx \end{array}$$

For *Pseudomonas* spp. and *S. cerevisiae*, the raw data recorded, according to the fixed experimental plan, were modeled using a software Package (Statistica for Windows, Statsoft, Tulsa, USA) to fit the second order model to dependent variables, i.e., the spoilage microorganism cell loads. The variables with a significance lower than 95% (*p* >0.05) were not included in the final models which were re-fitted after removing the non-statistically significant terms. The goodness of fit of the models obtained was evaluated using the Fisher *F*-test (and the derived *p*-values).

Three-dimensional surface plots were drawn to illustrate the major and interactive effects of the independent variables on the dependent ones. These graphs were drawn imposing a constant value (i.e., the central points of the interval taken into consideration) to one of the three independent variables.

The cell load data, recorded during the storage, ranging between 86.5 and 144.5 h, at the different temperatures of the CCD, for *Pseudomonas* spp. were also modeled using the Gompertz equation modified by Zwietering et al. (1990).

$$\begin{array}{l} y\;=\;K\;+\;A\;\times \text{ exp}\left\{-\text{exp}\left[\left(\mu \text{max }\times \;e/A\right)\;\times \;\left(ƛ-\text{t}\right)\;+\;1\right]\right\} \end{array}$$

where *k* is initial level of yeast/*Pseudomonas* (log cfu/fruit)

A is the maximum cellular density increase with respect to the initial cell load (k) (log cfu/fruit).

μmax: maximum specific growth rate (log (cfu/fruit)/hours).

λ: latency time (lag time) (hours).

*t* is the time (hours) necessary to reach the cell load of 7.0 log cfu/fruit chosen as arbitrary spoilage threshold. This value was used to solve the equation and calculate the time necessary to reach 7.0 log cfu/fruit.

At least 6 different cell loads recorded during the whole storage period (86.5–144.5 h) were used to obtain the kinetic parameters.

## Results and discussion

The quality of peaches, after the sanitizing treatment and before packaging, was checked evaluating the contamination level of *Pseudomonas* spp., yeasts and *E. coli* of 30 peaches randomly collected. All the microorganisms considered were under the detection limits (data not shown). On the other hand, the fruits used were un-ripened, sanitized and of high quality (without lesions).

### Transferring of *E. coli* from packaging material to fruits and probability of contamination packed fruits

The cell loads of *E. coli* recorded in the packed peaches, in relation to the considered run of the experimental design and to packaging materials used, are shown in Tables 2, 3. The data clearly showed a higher contamination frequency of the fruits packed in plastic than in cardboard boxes. In fact, *E. coli* was sporadically detected in runs 4, 8, 7, 15, 18, and 12, at levels ranging between 30 and 1920 cfu/fruit when cardboard wad used as packaging material. By contrast, the number of contaminated fruits and the cell loads per fruit dramatically increased when plastic boxes were used with the exceptions of the runs 4 and 19 where the contamination levels were under the detection limits. In fact, the percentage of samples positive for *E. coli* attained 90–95% of the analyzed fruits in 3 of the runs (5, 13, and 16) of the experimental design when the plastic was used as packaging materials, while it never exceeded 25% of fruits stored in cardboard. On the other hand the role of surfaces and inert materials to transfer, directly or indirectly microorganisms from a contaminated item to a non-contaminated one, is well known (Minnesota Department of Health, 2007; Cunningham et al., 2011; Erickson et al., 2015). In particular, Foong-Cunningham et al. (2012) underlined the importance of adequate cleaning and sanitization procedures to reduce the microbial contamination of fruit and vegetable RPC to improve the safety and shelf-life of fresh produces.

**Table 2:** *****Escherichia coli*** cell loads, expressed as cfu/fruit, recorded for each analyzed fruit (in total 20 for each run) packed in plastic material**.

**Run**	**T (°C)**	**Time (h)**	**Lesions (n)**																				
1	9	29	1	1980	–^*^	–	–	–	–	2160	–	–	–	9000	30	30	150	30	9000	60	1140	–	3000
2	9	58	1	–	–	–	–	150	–	–	–	–	–	–	–	–	60	–	–	90	–	–	–
3	9	29	3	930	9000	30	1800	30	30	9000	–	–	1800	–	60	1920	570	–	–	9000	90	180	90
4	9	58	3	–	–	–	–	–	–	–	–	–	–	–	–	–	–	–	–	–	–	–	–
5	19	29	1	9000	9000	9000	9000	9000	9000	–	30	3000	9000	9000	9000	750	1170	9000	1500	1650	300	120	450
6	19	58	1	–	–	–	–	30	30	30	30	90	30	180	90	360	450	30	–	180	–	150	60
7	19	29	3	–	–	60	180	30	60	30	60	9000	9000	300	1800	30	390	90	–	–	–	840	–
8	19	58	3	180	9000	270	–	60	–	–	–	–	300	–	210	–	660	–	–	630	9000	450	–
9	14	43.5	2	30	180	120	30	30	9000	9000	9000	60	90	90	1260	210	–	510	9000	9000	1020	1800	1680
10	14	43.5	2	9000	120	30	–	–	30	–	–	–	–	–	9000	390	210	–	9000	30	–	–	–
11	14	14.5	2	6030	–	–	660	–	4290	4500	–	690	30	4500	30	–	900	900	3150	9000	9000	4500	9000
12	14	72.5	2	30	150	60	–	–	–	30	9000	9000	30	–	–	–	–	1200	900	60	60	90	–
13	14	43.5	0	60	–	9000	9000	9000	9000	9000	–	30	90	210	180	1440	30	9000	1500	540	30	9000	840
14	14	43.5	4	–	30	–	–	360	30	9000	9000	450	9000	480	–	360	1110	2340	1680	9000	9000	9000	900
15	4	43.5	2	–	–	–	–	–	–	–	–	–	–	–	–	–	–	–	–	–	–	–	–
16	24	43.5	2	150	–	120	60	9000	9000	9000	9000	9000	–	540	9000	870	9000	1080	9000	9000	9000	9000	2100
17	14	43.5	2	–	90	30	60	60	–	–	9000	–	9000	–	120	450	210	240	450	–	–	570	2040
18	19	58	0	–	–	–	–	–	–	–	–	60	30	30	150	570	–	–	–	–	–	–	–
19	9	58	0	–	–	–	–	–	–	–	–	–	–	–	–	–	–	–	–	–	–	–	–
20	9	29	0	–	–	90	30	30	30	–	–	30	60	30	–	–	30	60	60	240	–	90	60

* *Under the detection limit (30 cfu/fruit)*.

**Table 3:** *****Escherichia coli*** cell loads, expressed as cfu/fruit, recorded for each analyzed fruit (in total 20 for each run) packed in corrugated cardboard boxes**.

**Run**	**T (°C)**	**Time (h)**	**Lesions (n)**																				
1	9	29	1	–^*^	–	–	–	–	–	–	–	–	–	–	–	–	–	–	–	–	–	–	–
2	9	58	1	–	–	–	–	–	–	–	–	–	30	30	–	–	–	–	–	–	–	–	–
3	9	29	3	–	–	–	–	–	–	–	–	–	–	–	–	–	–	–	–	–	–	–	–
4	9	58	3	–	30	–	–	–	–	–	–	–	–	–	–	–	–	–	–	–	–	–	–
5	19	29	1	–	–	–	–	–	–	–	–	–	–	–	–	–	–	–	–	–	–	–	–
6	19	58	1	–	30	–	–	–	90	–	–	–	–	–	–	–	–	–	–	–	–	–	–
7	19	29	3	–	–	–	–	–	–	–	–	–	30	90	30	–	30	–	–	–	–	–	–
8	19	58	3	–	–	–	–	30	–	–	–	–	–	–	–	–	–	–	–	–	–	–	–
9	14	43.5	2	–	–	–	–	–	–	–	–	–	–	–	–	–	–	–	–	–	–	–	–
10	14	43.5	2	–	–	–	–	–	–	–	–	–	–	–	–	–	–	–	–	–	–	–	–
11	14	14.5	2	–	–	–	30	–	–	–	–	–	–	–	–	–	30	–	–	–	30	–	–
12	14	72.5	2	–	–	–	–	270	30	–	–	–	–	–	–	570	60	30	–	–	–	–	–
13	14	43.5	0	60	30	90	–	–	–	–	–	–	–	–	–	30	30	–	–	–	–	–	–
14	14	43.5	4	–	–	–	–	–	–	–	–	–	–	–	–	–	–	–	–	–	–	–	–
15	4	43.5	2	–	1920	480	–	–	–	–	–	–	–	–	–	–	–	–	–	–	–	–	–
16	24	43.5	2	–	–	–	–	–	–	–	–	–	–	–	–	–	–	–	–	–	–	–	–
17	14	43.5	2	–	–	–	–	–	–	–	–	–	–	–	–	–	–	–	–	–	–	–	–
18	19	58	0	300	–	–	–	–	–	–	1440	30	–	–	–	–	–	–	–	–	–	–	–
19	9	58	0	–	–	–	–	–	–	–	–	–	–	–	–	–	–	–	–	–	–	–	–
20	9	29	0	–	–	–	–	–	–	–	–	–	–	–	–	–	–	–	–	–	–	30	–

* *Under the detection limit (30 cfu/fruit)*.

In this research, the logit model, based on the linearization of the logistic equation, was used to find relationships among the considered variables and the probability of transferring of *E. coli* from packaging materials to stored fruits, in relation to the independent variables adopted (temperature of storage, commercialization time, and number of fruit lesions). In fact, logit models are particularly useful when the observations to be modeled are not continuous but express the probability of an event (for example, growth/no growth and the presence or the absence of a specific microorganism). Based on empirical data, logistic regression calculates the probability of a binary outcome as a linear function of a combination of predictor variables (Hosmer and Lemeshow, 1989). The application of logistic models were applied to predict bacterial and yeast growth in several food matrices (Lanciotti et al., 2001; Membre et al., 2001; Koutsoumanis et al., 2004; Belletti et al., 2007, 2010; Dang et al., 2010). In order to develop the model the value 0 was assigned to the absence of *E. coli* while 1 was assigned to the presence of *E. coli* on fruit surface.

The equations obtained in relation to packaging material used and the statistical diagnostics (Chi square and P) are reported in Table 4.

**Table 4:** **Coefficients estimated for the polynomial equations obtained for plastic and corrugated cardboard boxes**.

	**Plastic boxes**	**Corrugated cardboard boxes**
Costant	0.176	−4.73
Commercialization time	−0.0587	0.019
Number of lesions	0.11	−0.179
Storage temperature	0.1917	0.103
Chi-square	103.46	9.33
	*P* < 0.00001	*P* = 0.025

**Figure 1:**
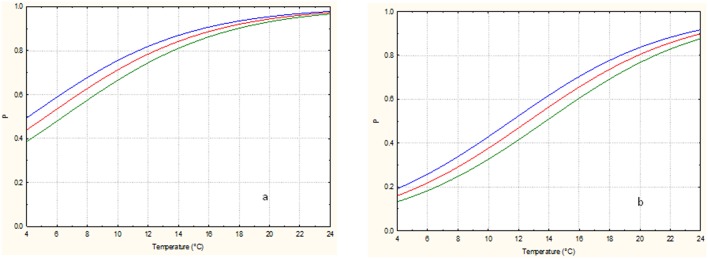
*****Escherichia coli*** transfer probability from plastic to packed peaches after 24 (a) and 48 (b) hours of commercialization in relation to the storage temperature and number of lesions**. The green, red and blue lines correspond to 0, 2, and 4 lesions, respectively.

**Figure 2:**
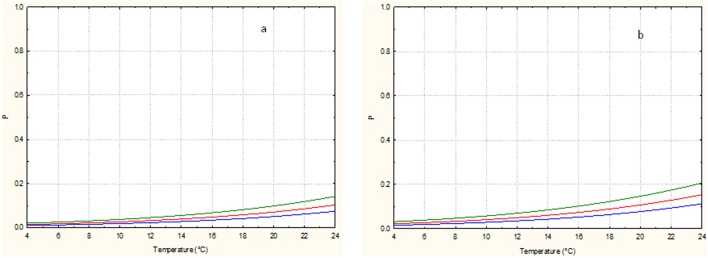
*****Escherichia coli*** transfer probability from corrugated cardboard boxes to packed peaches after 24 (a) and 48 (b) hours of commercialization in relation to the storage temperature and number of lesions**. The green, blue and red lines correspond to 0, 2, and 4 lesions, respectively.

To better pinpoint the effect of the independent variables taken into consideration on the *E. coli* transferring probability in relation to packaging material, Figures 1, 2 where drown from the equation of Table 4, maintaining the commercialization time to a fix value of the experimental design (24 or 48 h). Increasing the storage temperature and the number of fruit lesions, the probability of transferring of *E. coli* from packaging materials to fruits increased, independently on commercialization time or type of material used. The number of lesions showed an highest effect on fruits stored at lowest temperatures compared to those stored at the highest ones of the experimental design. In any case, the probability of *E. coli* transfer from packaging to packed peaches is higher in plastic material.

The comparison showed clearly the negative effects of commercialization time on the transferring probability of *E. coli* when the fruits were stored at low temperature. This negative effect can be attributed to the incapability of the strain used to growth at 4–8°C. Otherwise the literature data showed that *E. coli* can remain viable on several types of surfaces for long period (up to some days) in appropriate physic-chemical conditions (in terms of humidity, temperature, atmosphere composition) and nutrient availability (DeVere and Purchase, 2007). It is well known that several extrinsic (environmental) and intrinsic factors can contribute to the dynamics of pathogen transference as showed by Pérez-Rodríguez et al. (2008). Among the extrinsic factors the surface properties and level of moisture, the relative humidity in the atmosphere, and the time of contact (i.e., the commercialization time) were indicated as the most significant ones. Among the intrinsic factors, the presence of exopolysaccharides and the pathogen contamination level were the most important for the microbial transferring.

### Transferring of *Pseudomonas* and *S. cerevisiae* in relation to packaging material and effects on fruit shelf-life

**Figure 3:**
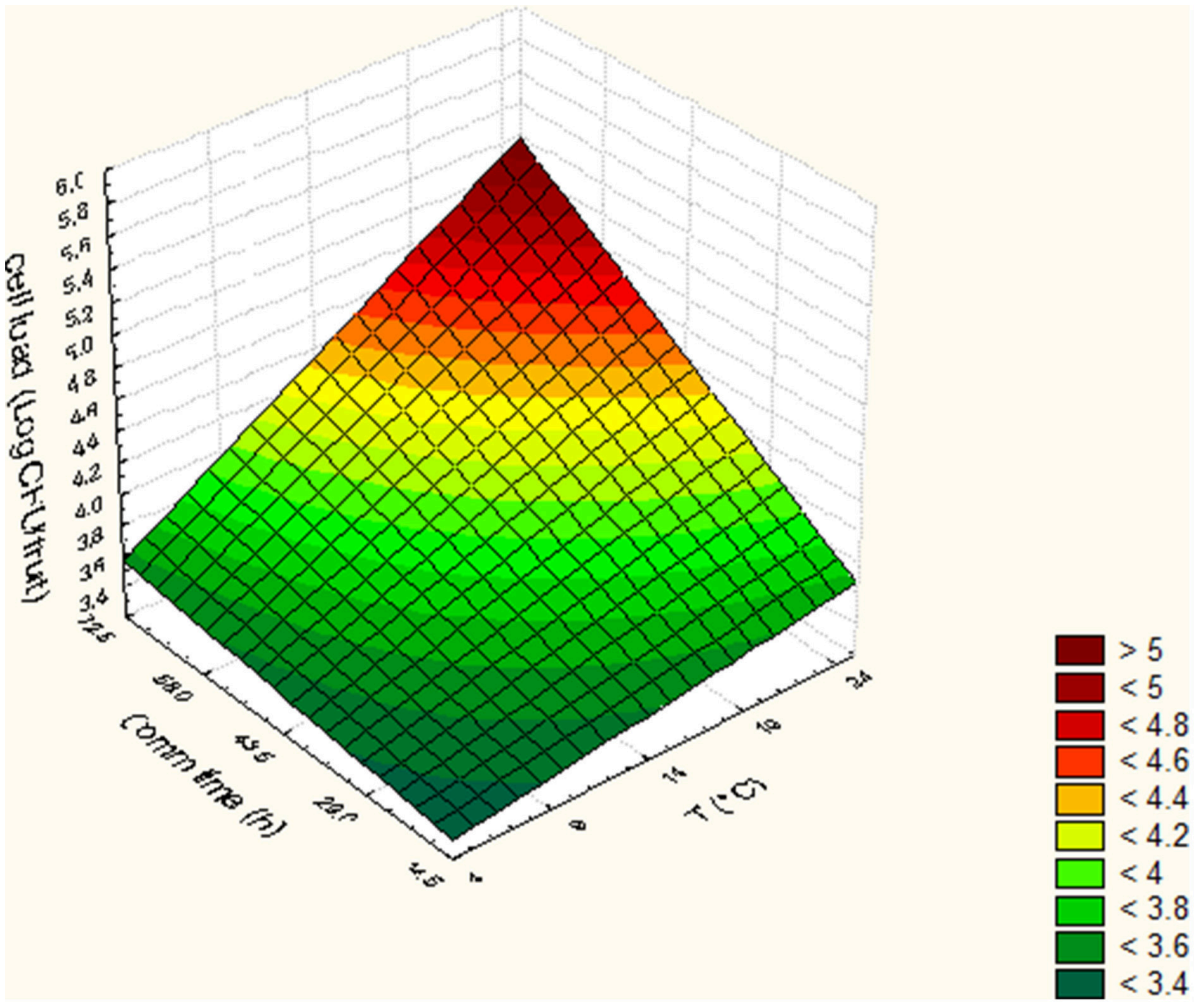
*****Saccharomyces cerevisiae*** cell loads (log cfu/fruit) detected in peaches packed in plastic in relation to the Temperature and commercialization time**.

**Figure 4:**
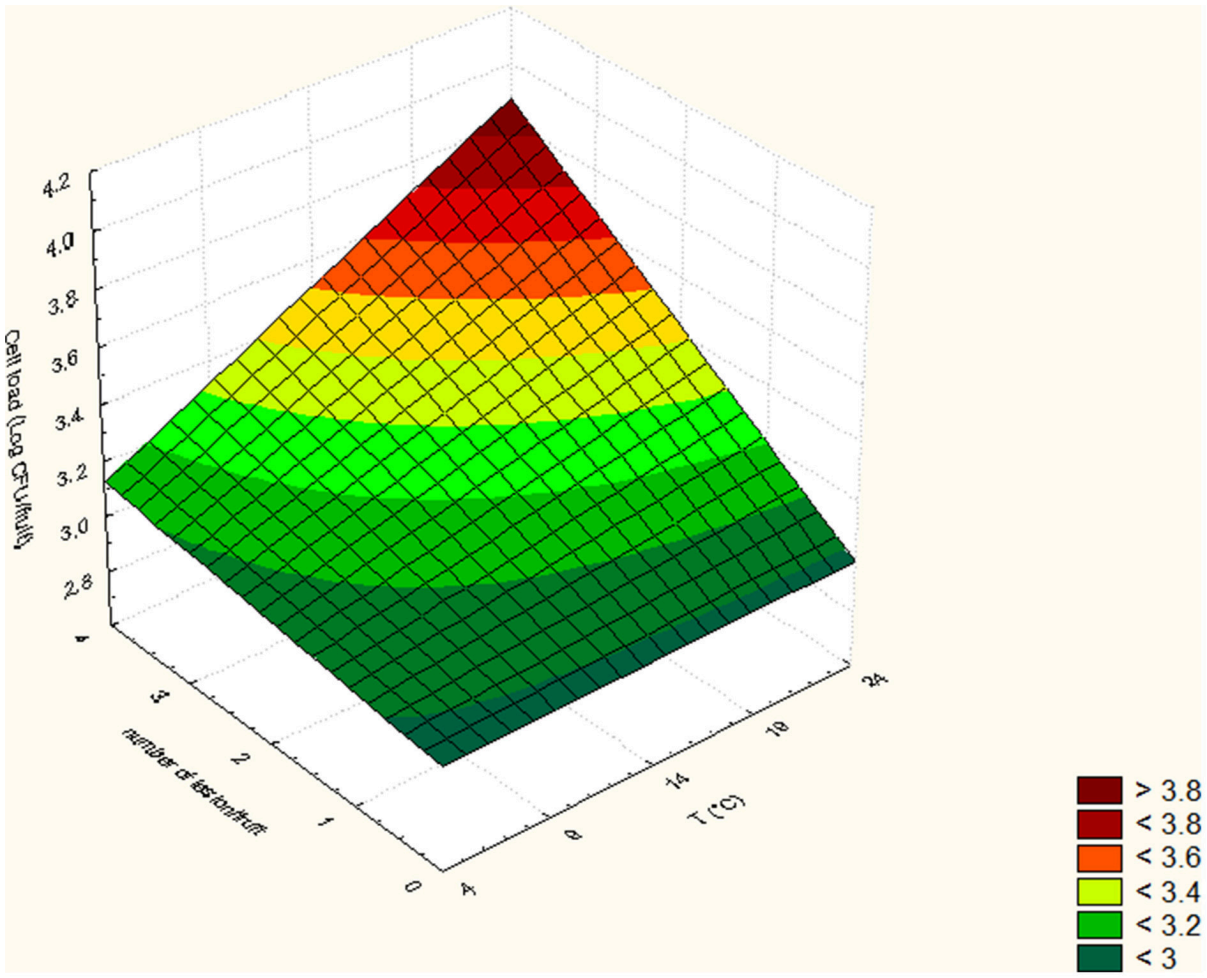
*****Saccharomyces*** cell loads (log cfu/fruit) detected in peaches packed in corrugated in relation to the Temperature and number of lesion**.

**Figure 5:**
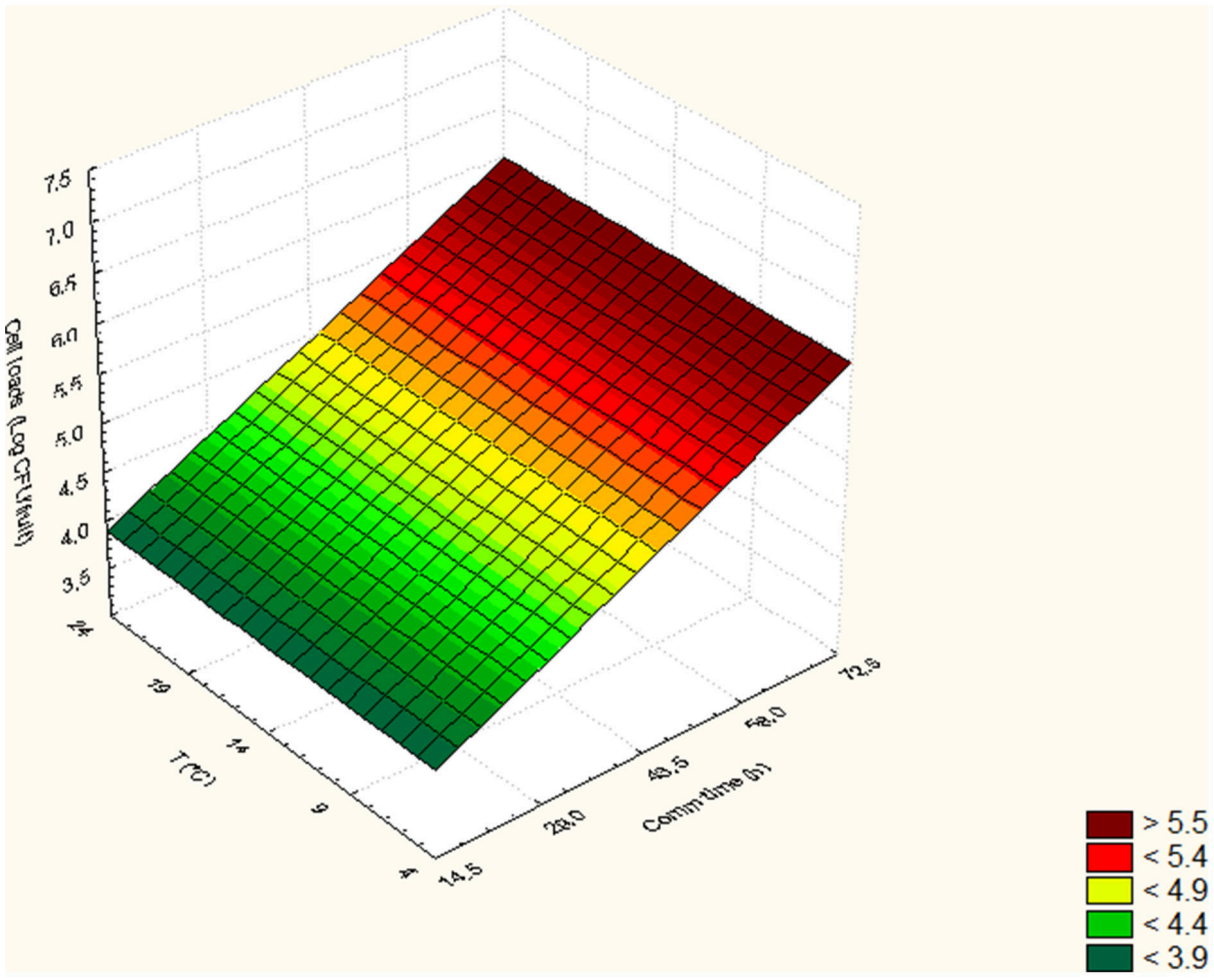
*****Pseudomonas*** cell loads (log cfu/fruit) detected in peaches packed in plastic in relation to the Temperature and commercialization time**.

**Figure 6:**
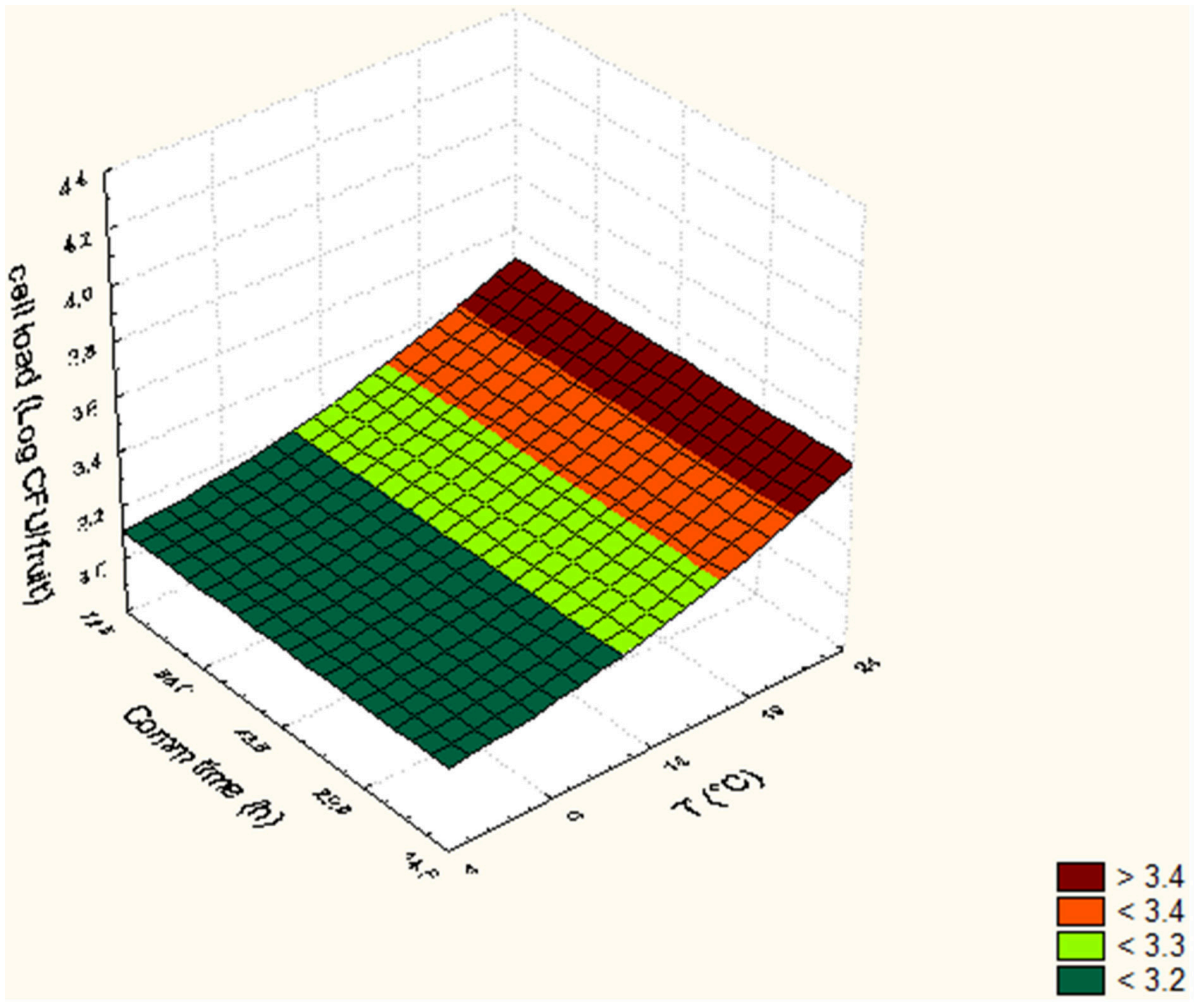
*****Pseudomonas*** cell loads (log cfu/fruit) detected in peaches packed in corrugated in relation to the Temperature and commercialization time**.

**Figure 7:**
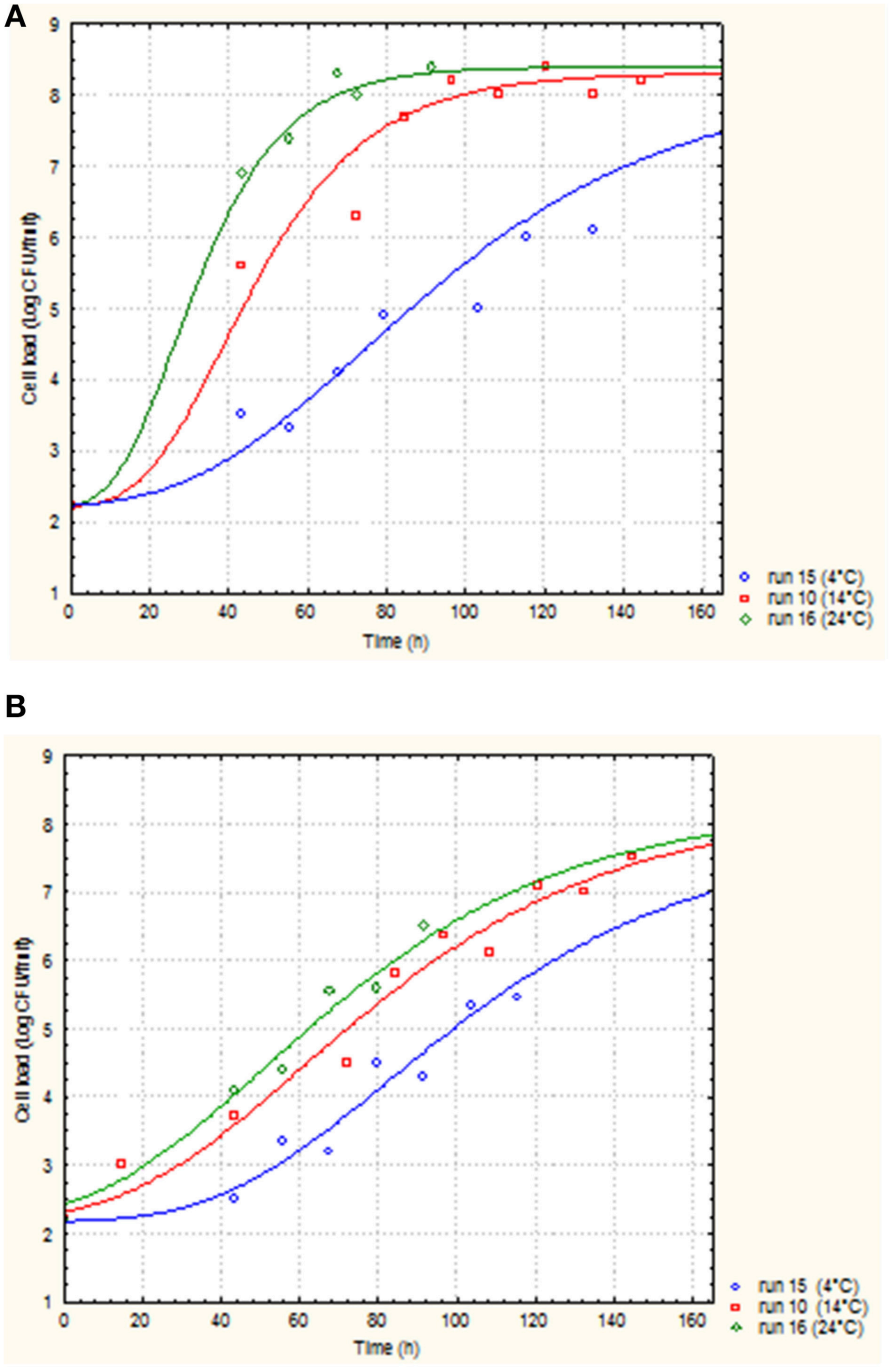
**Experimental data points of ***Pseudomonas*** spp. recorded in peaches stored in plastic (A) and in cardboard (B) at 4°C (run 15, blue line) 14°C (run 10, red line), 24°C (run 16, green line) obtained from Gompertz model fitting**.

In Tables 5, 6, the cell loads detected for *Pseudomonas* in the different runs of the experimental design in relation to packaging material used are reported. The contamination levels detected on fruits packaged in plastic boxes were significantly higher compared to those found on fruits stored in cardboard boxes, independently on the level of the independent variables considered (temperature, commercialization time, and number of lesions). In fact, in the peaches packed in corrugated boxes, the contamination levels of *Pseudomonas* spp. were 1.0 log lower than those detected on fruits stored in plastic. Also for *S. cerevisiae*, significantly lower contamination levels were detected in peaches stored in corrugated compared to those packed in plastic boxes. In particular, the peaches stored in plastic showed yeast cell loads higher than those stored in corrugated of at least 1.5 log cfu/fruit. However, differences up to 2.8 log cfu/fruit (i.e., run 18) were found in relation to the packaging used. The RSM was used to study the main, interactive and quadratic effects of storage temperature, commercialization time and number of lesions on the cell loads and shelf-life of the fruits in relation to packaging material used. RSM is a statistical tool, which is used to design the experiments, build models, thereby, evaluate the effect of various variables on one or more responses and sets an optimal solution for the responses with reduction in the number of experimental runs (Bas and Boyaci, 2007; Uncu and Cekmecelioglu, 2011). Most commonly used design in RSM is Central Composite Design (CCD) which is characterized by flexible rotation in the design space, more precise predictions about the response of the variables along with the information about the experimental errors (Montgomery, 2009). RMS and CCD were widely used to study microbial growth parameters and/or food microbial shelf-life in relation to several physic-chemical, process and storage conditions (Lanciotti et al., 1999; Patrignani et al., 2006, 2007; Bas and Boyaci, 2007; Uncu and Cekmecelioglu, 2011; Arora et al., 2015). The polynomial equations obtained modeling the fruit cell load data of *Pseudomonas* and *S. cerevisiae*, in the different runs of the CCD, in relation to packaging material used are reported in Table 7. The polynomial equations showed that the cell load of *S. cerevisiae* of fruits stored in plastic boxes was positively affected by the quadratic term of temperature and by the interaction between temperature and commercialization time. The effects on storage temperature and commercialization time on the cell loads of *S. cerevisiae* is better showed by Figure 3, obtained from equation 1 maintaining the lesion number on its central value of the CCD (2 lesions). In fact, in the fruits with 2 lesions *S. cerevisiae* cell loads reached the maximum level after 72.5 h of storage at 24°C. According to equation 2 of Table 7, *S. cerevisiae* cell load was positively and significantly affected by the interaction between temperature and number of lesions. As shown by Figure 4, obtained maintaining at its central value the commercialization time, the highest levels of *S. cerevisiae* were observed in peaches having 4 lesions and stored at 24°C. *S. cerevisiae* growth in fruits packed in cardboard boxes was reduced compared with that of fruits packed in plastic during the storage due to the reduced transferring from packaging materials to peaches. The positive effect of the number of lesions on the cell loads of *S. cerevisiae* was due to the release of cell content from damaged tissue that is reported to stimulate the microbial growth (Lanciotti et al., 2004b; Siroli et al., 2014; Patrignani et al., 2015). As shown by equation 3 and 4 and Figures 5, 6, the growth of *Pseudomonas* spp., microorganism notoriously endowed with reduced nutritional requirements compared to *S. cerevisiae*, was positively affected by the commercialization time and storage temperature in plastic and cardboard boxes, respectively. The significantly lower cell loads recorded in fruits stored in cardboard resulted in significant reduction of the growth potential of the spoilage microorganisms taken into consideration. In fact, although the microbial growth is only one of the several factors affecting fresh produce, the time necessary to reach 7 log ufc/fruit by *Pseudomonas* spp. in peaches stored in cardboard was 24 and 72 h longer than that in plastic and this time was taken as an arbitrary measure of shelf-life in our experimental condition (Tables 5, 6). The time necessary to reach the threshold was calculated according to Gompertz equation that fitted well the experimental data recorded over time as shown by the Figures 7A,B, relative to peaches, having the same number of lesions, stored at 24, 14, and 4°C, in plastic and corrugated, respectively. In fact, in the experimental plan used the number of lesions was an independent variable. It is well known that the wounds are points in which the microbial multiplication is increased and in our experimental conditions the threshold value of 7 log cfu/fruit was associated to an evident fruit sensory spoilage. The delay of microbial growth is important not only for its effect on fruit microbiological quality and shelf-life but also because it meets the consumers' expectation, preferences and habits. In fact, the attractiveness of fresh produces for consumers is determined also by organoleptic factors like appearance, firmness, taste and perceived health benefits as well as by safety and shelf-life of the product (Malmendal et al., 2011; Cuthbertson et al., 2012; Santucci et al., 2015). The fruit considered in this research (peach), being a living organism with high metabolic activity, is subjected to a rapid quality decreases after harvest due mainly to ethylene production. This causes several negative effects including senescence, accelerated quality loss, reduced nutrient composition, increased fruit pathogen susceptibility, physiological disorders in fruit and vegetables, and consequently the growth potential of microorganisms present on fruit surfaces (Martínez-Romero et al., 2007; Liu et al., 2015). Microbial growth can significantly affect fruit shelf-life and, in the case of pathogenic species, fresh produce safety features. Consequently, the reduction of the transferring of the microorganisms from the packaging materials, through the choose of the type of materials, can represent an important strategy to increase food safety, shelf-life and sensory features as well as the sustainability of the whole fresh produce production and distribution chain (decrease of waste, water and energy consumption). In conclusion, the data obtained showed that cardboard compared to plastic can significantly reduce the potential of packaging material to act as microorganism source for cross contamination of fresh produces. In fact, both the probabilistic (logit model) and the deterministic models (according to RSM) showed a higher microbiological qualities (in terms of transferring probability for *E. coli* or cell load recorded for *S. cerevisiae* and *Pseudomonas* spp.) of peaches stored in cardboard boxes independently on the independent variables considered. In addition, the mathematical approaches used permitted also to evaluate the role of the temperature, commercialization time and number of lesions on the microbial transferring and the fruit microbiological quality of the product independently to packaging material used. Consequently, this data can contribute to optimize the fresh produce logistic and distribution, even if the model needs to be validated by a scaled-up trial. The best performances of cardboard compared to plastic presumably can be due to the reduction of the superficial contamination level of corrugated cardboard boxes compared to plastic due to its entrapping capability in the fiber of cardboard. However, although the results of this study indicate the use of cardboard as a tool to reduce the microbial contamination level of fresh produces, further studies are necessary to verify the entrapping capability of packaging material in relation to the storage and distribution conditions.

**Table 5:** *****Pseudomonas*** spp. cell loads, expressed as log cfu/fruit, of peaches packed in plastic in relation to the considered run and level of the independent variables**.

**Run**	Cell load^*^ (Log cfu/fruit)	Time to reach 7 log cfu/fruit^**^ (h)	**Temperature (°C)**	**Commercialization time (h)**	**Number of lesion (n)**
1	4.0	109	9	29	1
2	4.9	111	9	58	1
3	4.5	95	9	29	3
4	5.6	95	9	58	3
5	4.3	94	19	29	1
6	5.3	99	19	58	1
7	3.5	91	19	29	3
8	5.5	94	19	58	3
9	5.6	92	14	43.5	2
10	6.1	80	14	43.5	2
11	4.3	83	14	14.5	2
12	6.3	92	14	72.5	2
13	3.9	121	14	43.5	0
14	4.6	104	14	43.5	4
15	3.3	143	4	43.5	2
16	6.9	49	24	43.5	2
17	6.1	66	14	43.5	2
18	4.7	102	19	58	0
19	5.5	98	9	58	0
20	4.4	81	9	29	0

* *Cell loads at the commercialization time fixed by CCD*.

** *Time in hours necessary to reach 7 log cfu/fruit. This value was chosen because in our experimental conditions, it corresponds to a sensory spoilage and rejection of fruit*.

**Table 6:** *****Pseudomonas*** spp. cell loads, expressed as log cfu/fruit, of peaches packed in corrugated in relation to the considered run and level of the independent variables**.

**Run**	Cell loads^*^ (Log cfu/fruit)	Time to reach 7 log cfu/fruit^**^ (h)	Shelf-life increase^***^ (h)	**T (°C)**	**Commercialization time (h)**	**Number lesion (n)**
1	3.2	141	32	9	29	1
2	3.6	148	37	9	58	1
3	3.5	139	44	9	29	3
4	3.1	151	56	9	58	3
5	3.3	118	24	19	29	1
6	3.6	132	33	19	58	1
7	3.5	117	26	19	29	3
8	3.7	138	44	19	58	3
9	3.7	126	34	14	43.5	2
10	3.7	133	53	14	43.5	2
11	3.0	116	33	14	14.5	2
12	4.2	144	52	14	72.5	2
13	3.3	149	28	14	43.5	0
14	3.6	134	30	14	43.5	4
15	2.9	167	24	4	43.5	2
16	4.1	121	72	24	43.5	2
17	3.3	129	63	14	43.5	2
18	3.6	140	38	19	58	0
19	3.6	155	57	9	58	0
20	3.8	139	58	9	29	0

* *Cell loads at the commercialization time fixed by CCD*.

** *Time in hours necessary to reach 7 log cfu/fruit. This value was chosen because in our experimental conditions, it corresponds to a sensory spoilage and rejection of fruit*.

*** *Shelf-life increase of peaches packed in corrugated with respect ones placed in plastic*.

**Table 7:** **Best-fit equations relative to the effects of the different CCD independent variables on the ***Pseudomonas*** and ***Saccharomyces cerevisiae*** cell loads on peaches packed in plastic and corrugated cardboard boxes**.

Parameters^**^					
**Packaging**	Equation^*^	***R***	***F***	***df***	***p***
Plastic	*S. cerevisiae cell load* = 3.96+0.001294T^2^ + 0.001043T × t	0.91	43.91	2.18	0.000000
Corrugated	*S. cerevisiae cell load* = 2.976+0.00939T × nl	0.61	10.98	1.18	0.003866
Plastic	*Pseudomonas* spp. *cell load* = 3.37+0.035965t	0.53	7.27	1.18	0.014765
Corrugated	*Pseudomonas* spp. *cell load* = 3.09+0.000693T^2^	0.66	13.91	1.18	0.0015

* *Cell load expressed as log cfu/fruit; T = temperature in °C; t = commercialization time in hours; nl = number of lesion/fruit*.

** *R = regression coefficient; F = F-value; df = degree freedom; p = only terms with p < 0.05 were included*.

## Author contributions

RL and FP planned the experimental plan. FP and LS planned the lab activities. FG performed the analysis of the data and elaboration of the models.

### Conflict of interest statement

The authors declare that the research was conducted in the absence of any commercial or financial relationships that could be construed as a potential conflict of interest.
